# Computational ECG mapping and respiratory gating to optimize stereotactic ablative radiotherapy workflow for refractory ventricular tachycardia

**DOI:** 10.1016/j.hroo.2021.09.001

**Published:** 2021-09-20

**Authors:** Gordon Ho, Todd F. Atwood, Andrew R. Bruggeman, Kevin L. Moore, Elliot McVeigh, Christopher T. Villongco, Frederick T. Han, Jonathan C. Hsu, Kurt S. Hoffmayer, Farshad Raissi, Grace Y. Lin, Amir Schricker, Christopher E. Woods, Joey P. Cheung, Al V. Taira, Andrew McCulloch, Ulrika Birgersdotter-Green, Gregory K. Feld, Arno J. Mundt, David E. Krummen

**Affiliations:** ∗ Department of Medicine-Cardiology, University of California San Diego, La Jolla, California; † Department of Radiation Medicine, University of California San Diego, La Jolla, California; ‡ Department of Bioengineering, University of California San Diego, La Jolla, California; § Vektor Medical Inc, Carlsbad, California; ‖ Department of Pathology, University of California San Diego, La Jolla, California; ¶ Department of Cardiac Electrophysiology, Mills-Peninsula Medical Center, Sutter Health, Burlingame, California; # Department of Radiation Oncology, Mills-Peninsula Medical Center, Sutter Health, Burlingame, California

**Keywords:** Ablation, Cardiac computed tomography, Electrocardiography, Noninvasive mapping, Stereotactic ablative radiotherapy, Ventricular tachycardia

## Abstract

**Background:**

Stereotactic ablative radiotherapy (SAbR) is an emerging therapy for refractory ventricular tachycardia (VT). However, the current workflow is complicated, and the precision and safety in patients with significant cardiorespiratory motion and VT targets near the stomach may be suboptimal.

**Objective:**

We hypothesized that automated 12-lead electrocardiogram (ECG) mapping and respiratory-gated therapy may improve the ease and precision of SAbR planning and facilitate safe radiation delivery in patients with refractory VT.

**Methods:**

Consecutive patients with refractory VT were studied at 2 hospitals. VT exit sites were localized using a 3-D computational ECG algorithm noninvasively and compared to available prior invasive mapping. Radiotherapy (25 Gy) was delivered at end-expiration when cardiac respiratory motion was ≥0.6 cm or targets were ≤2 cm from the stomach.

**Results:**

In 6 patients (ejection fraction 29% ± 13%), 4.2 ± 2.3 VT morphologies per patient were mapped. Overall, 7 out of 7 computational ECG mappings (100%) colocalized to the identical cardiac segment when prior invasive electrophysiology study was available. Respiratory gating was associated with smaller planning target volumes compared to nongated volumes (71 ± 7 vs 153 ± 35 cc, *P* < .01). In 2 patients with inferior wall VT targets close to the stomach (6 mm proximity) or significant respiratory motion (22 mm excursion), no GI complications were observed at 9- and 12-month follow-up. Implantable cardioverter-defibrillator shocks decreased from 23 ± 12 shocks/patient to 0.67 ± 1.0 (*P* < .001) post-SAbR at 6.0 ± 4.9 months follow-up.

**Conclusions:**

A workflow including computational ECG mapping and protocol-guided respiratory gating is feasible, is safe, and may improve the ease of SAbR planning. Studies to validate this workflow in larger populations are required.

## Graphical abstract

**Figure:**
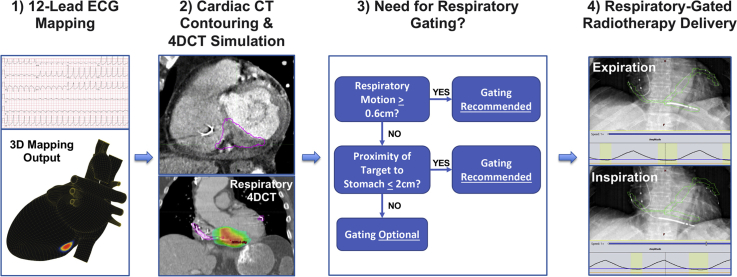

**Key Findings**

▪ Automated computational 12-lead electrocardiogram mapping is feasible and may facilitate target planning via 3-D visualization of ventricular tachycardia (VT) exit sites with precision appropriate for noninvasive VT ablation.
▪ Respiratory motion can range up to 22 mm; protocol-guided respiratory-gated delivery of radioablation appears feasible and may improve treatment precision in patients with significant respiratory motion.
▪ The inferior left ventricle is a high-risk region for stereotactic ablative radiotherapy therapy owing to its proximity to the stomach (down to 4 mm proximity), providing additional rationale for respiratory gating in patients with VT sources in this region.

## Introduction

Refractory ventricular tachycardia (VT) is a life-threatening condition that may cause recurrent implantable cardioverter-defibrillator (ICD) shocks and progressive heart failure. Standard therapies include antiarrhythmic medications, invasive catheter or surgical ablation, and autonomic modulation.^1^, ^2^, ^3^ When these therapies fail or if the patient is not a candidate for further invasive therapies, limited options exist.

Stereotactic ablative radiotherapy (SAbR) has been reported for refractory VT in several centers, with promising efficacy and low acute complication rates.^4^, ^5^, ^6^, ^7^, ^8^ Arrhythmia source mapping for SAbR has been performed based upon either the results of prior invasive electrophysiology study, electrocardiographic imaging (ECGi), or manual assessment of the ECG.^4^^,^^6^^,^^7^^,^^9^^,^^10^ However, each of these methods may be suboptimal owing to logistical requirements or limitations to mapping accuracy.

Prior studies have primarily delivered SAbR without respiratory gating or with abdominal compression to dampen respiratory motion, but active respiratory gating using optical body surface tracking to compensate for respiratory motion during radiotherapy delivery has not been previously described.

We hypothesized that a novel SAbR workflow using computational ECG mapping combined with protocol-guided, respiratory-gated radiation delivery is feasible, is safe, and may facilitate the treatment of inferior left ventricular VT targets. We aim to (1) assess the efficacy of this workflow in reducing ICD shocks as a primary endpoint, (2) demonstrate the feasibility of novel computerized ECG mapping to guide noninvasive target planning, and (3) evaluate the feasibility and precision of a novel protocol-directed respiratory-gating strategy to minimize respiratory motion, particularly at inferior wall targets close to the stomach.

## Methods

### Patient selection

We studied consecutive patients with refractory VT in 2 tertiary medical systems (University of California San Diego, La Jolla, CA, and Mills-Peninsula Medical Center, Sutter Health, Burlingame, CA) undergoing noninvasive radioablation after failing all available therapies including antiarrhythmic medications, catheter ablation, and stellate ganglion blockade who were not candidates for additional ablation attempts or cardiac transplantation.

The workflow included use of a computational ECG mapping algorithm and protocol-guided respiratory-gated therapy when cardiac respiratory motion was ≥0.6 cm or VT sites of origin ≤2.0 cm from the stomach. The study was performed in accordance with an institutional review board–approved protocol and adhered to the Helsinki guidelines; all patients provided written informed consent.

### Computerized ECG analysis and visualization

Patients underwent noninvasive programmed stimulation (NIPS) using their ICD (details in Supplemental Appendix, section I) and recorded by an electrophysiology recording system. ECG data were analyzed using a proprietary computational ECG mapping algorithm (written in Python; Python Software Foundation, Beaverton, OR) and displayed on a 3-D heart model (Blender; Blender Foundation, Amsterdam, Netherlands).^11^ The automated mapping algorithm analyzes the vectorcardiographic data from the patient’s 12-lead ECG and computes the probabilistic locations derived from electroanatomic biventricular computer models. Patient-specific factors such as location of scar, ventricular dilation, and hypertrophy are factored into the mapping process, if such data are available. The VT exit sites are also automatically assigned the corresponding cardiac segments within both the left (LV) and right ventricles (RV), based on prior work using a 30-segment ventricular model.^12^ Further details describing the algorithm are located in section I of the Supplemental Appendix.

### Mapping assessment and comparison to manual QRS morphology algorithm

When available, computed VT exit sites were compared with the results of invasive electrophysiology study from prior unsuccessful catheter ablation attempts.

Accuracy of the computational ECG algorithm was assessed by determining if the computed VT exit sites were in the same cardiac segment (using the 30-segment ventricular model) as the VT exit sites by invasive mapping for matched VT morphologies (same QRS morphology in 12 of 12 ECG leads).^12^

For comparison, we also analyzed VT QRS morphologies using a contemporary manual QRS morphology algorithm by Andreu and colleagues^13^ and determined the segment of origin for each VT.

## SAbR therapy planning

Three-dimensional maps displaying the VT exit sites were exported for therapy planning (Graphical Abstract, left panel). High-resolution (0.625 × 0.5 × 0.5 mm), dose modulated, cardiac-gated computed tomography (CT) scans (Revolution CT; GE Healthcare, Chicago, IL) were obtained to identify potential arrhythmogenic substrate, defined as wall thinning <5 mm (Supplemental Figures S1–S3).^14^ Data from other imaging modalities including cardiac magnetic resonance imaging (MRI), nuclear sestamibi, and prior voltage mapping were also integrated into a comprehensive assessment of LV substrate, and final target volumes were contoured onto the cardiac CT and then transferred onto the radiation simulation CT per protocol (Graphical Abstract 1, middle left panel). Efforts were made to integrate target regions for VTs exiting from a common central scar to create a continuous lesion, anchored to nonconducting structures when feasible.

### Preprocedural assessment of respiratory motion

Both the magnitude of cardiac respiratory motion and proximity of the VT target to the gastrointestinal (GI) system were assessed using both the simulation 4D-CT and NIPS fluoroscopy to determine the need for respiratory gating. Respiratory gating was used when either the proximity of the VT target to a GI structure was ≤2.0 cm or respiratory motion of the closest intracardiac fiducial (ICD or coronary sinus [CS] lead tip) was ≥0.6 cm in the cranial-caudal axis during normal breathing (Graphical Abstract, middle right panel). These cutoff values were derived from prior studies analyzing ranges of cardiac motion during respiration.^15^ The full respiratory-gating protocol is detailed in the Supplemental Appendix, Section II.

### Respiratory gating during SAbR delivery

For patients assigned to respiratory gating, radiation therapy was delivered during a prespecified window at end-expiration using an external optical surface tracking system to evaluate thoracic motion (AlignRT; Vision RT, London, UK).

For patients not assigned to respiratory gating, therapy was delivered throughout the respiratory cycle (free-breathing). In both groups, radiation therapy was interrupted during irregular respiration patterns (eg, large inspiration, coughing).

Fluoroscopic imaging was used intermittently to verify that the fiducials were correctly aligned with the planning fiducial contours drawn on simulation scan. The fiducials used in this study were radiographic features such as an ICD or CS lead tip used to help confirm the alignment of the patient at end-expiration. We used the ICD lead tip as the primary fiducial for all study patients and the CS lead tip as a secondary fiducial in a subset of patients.

Radiation (6 MV, 25 Gy) was delivered in a single fraction using a linear accelerator (TrueBeam; Varian, Palo Alto, CA). Details of retrospective respiratory and cardiac cycle motion analysis is included in the Supplemental Appendix, sections III and IV.

### Procedural ICD programming

At NIPS and the time of SAbR therapy, VT treatment zones were adjusted with lowest zone set 10 beats per minute below slowest clinical VT cycle length, as previously described.^14^ Baseline programming was restored immediately following SAbR delivery. Antiarrhythmic medications were tapered at the discretion of the treating electrophysiologist.

### Clinical follow-up

The primary endpoint of the study was all-cause ICD shocks pre- and post-SAbR, including a 6-week blanking period. Patients were followed in clinic at 1, 3, 6, and 12 months after treatment with device interrogations at each visit to allow quantification of VT burden and secondary safety endpoints (pericarditis, pericardial effusion, pleural effusion, accelerated valvular disease, GI injuries such as gastropericardial fistula, myocardial infarction, skin burns, allergic reaction, and death). Echocardiograms and short quality-of-life assessment (CCS-SAF) were performed prior to therapy and at 1 and 6 months; chest CT scans were obtained based on changes in pulmonary symptoms.^16^

### Statistical analysis

Data are reported as mean ± standard deviation for normally distributed data or median [interquartile range] for non-normally distributed data. Fisher exact test was used to compare proportions between groups; the paired *t* test was used to compare within-patient data. The McNemar test with continuity correction was used to assess agreement between the mapping algorithm and prior electroanatomic mapping. Comparisons were 2-tailed; *P* < .05 was considered statistically significant. Statistics were calculated using SPSS version 27 (IBM, Armonk, NY).

## Results

We enrolled 6 patients (aged 74 ± 6.1 years, ejection fraction 29% ± 13%) refractory to 2.2 ± 1.1 antiarrhythmic drugs and 2.2 ± 1.0 ablation procedures. Table 1 and Supplemental Table S1 show the study population demographics and VT burden prior to SAbR therapy.

**Table 1:** Study Patient Demographics

	Patient 1	Patient 2	Patient 3	Patient 4	Patient 5	Patient 6
Age (years)	76	77	69	81	66	64
Sex	M	M	M	M	M	M
LV EF (%)	23	40	46	27	10	25
Cardiomyopathy type	NICM	ICM	NICM	NICM	ICM	NICM
NYHA class	IV	III	III	IV	IV	IV
Failed AAD, n	4	2	2	2	1	5
Failed catheter ablations, n	3	3	3	1	1	2
Preablation ICD shocks, n	33	24	34	28	7	10
Distinct VTs induced, n	6	4	2	1	5	7
VT locations	Basal septum, LV summit, anterior mitral annulus	Epicardial basal inferoseptal RV and LV (crux)	Mid septum	Mid-anterolateral LV	Inferior mid and apical LV	LV summit, anterior mitral annulus
VT cardiac segment†	1, 2, 6	3, 4, 23	8, 9	12	15, 17	1, 2, 6
Scar location	Basal septum, perimitral	Inferior basal RV and LV	Septum	Inferolateral, anterolateral LV	Inferior wall	Basal anterior, anterolateral LV

AAD = antiarrhythmic drug; EF = ejection fraction; ICD = implantable cardioverter-defibrillator; ICM = ischemic cardiomyopathy; LV = left ventricle; NICM = nonischemic cardiomyopathy; RV = right ventricle; VT = ventricular tachycardia.

† Based on the 30-segment biventricular heart model (Plasier et al^12^).

### Clinical outcomes

**Figure 1:**
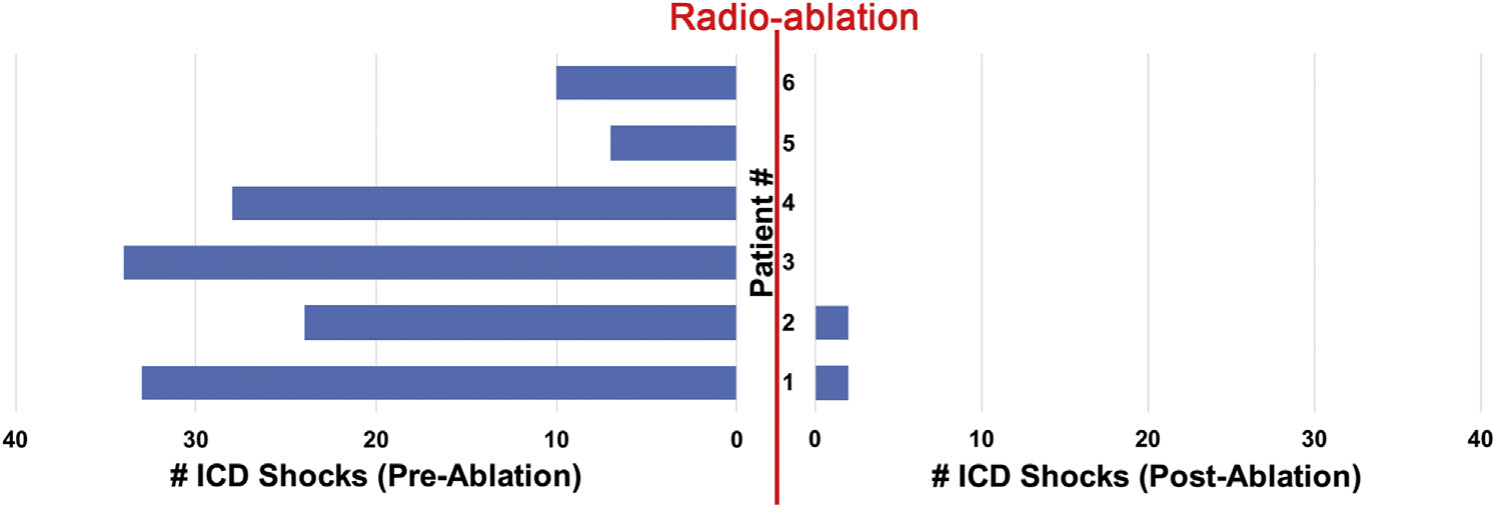
Reduction in implantable cardioverter-defibrillator (ICD) shocks following radioablation. ICD shocks were significantly decreased from 23 ± 12 shocks in the 6 months prior to stereotactic ablative radiotherapy (36 patient-months) to 0.67 ± 1.0 shocks at 6.0 ± 4.9 months follow-up (*P* < .005).

Analysis of the primary endpoint found that ICD shocks were significantly decreased from 23 ± 12 shocks per patient in the 6 months prior to SAbR therapy (36 patient-months) to 0.67 ± 1.0 shocks per patient at 6.0 ± 4.9 months follow-up (*P* < .005, Figure 1). Overall shock density decreased from 3.8 shocks/months before SAbR to 0.1 shocks/month after SAbR: a relative reduction of 97% from baseline. Temporally, 2 out of the 4 post-SAbR shocks occurred at week 2 during the 6-week blanking period in patient 1, while the other 2 shocks occurred for VF in patient 2 3 months after therapy without additional shocks in 12-month follow-up.

There was significant quality-of-life improvement from before treatment to 1 month follow-up; CCS-SAF score was reduced from 3.7 ± 0.5 to 2.8 ± 1.2, *P* = .04). Mean NYHA class improved from 3.7 ± 0.5 to 3.0 ± 0.9, *P* = .02) at 1 month. All patients remained on antiarrhythmic medications at last follow-up, with reduced doses of amiodarone in 2 patients (patients 2 and 4).

### Safety of SAbR

#### Mortality

Two patients died after treatment for reasons thought to not be directly related to radiotherapy. Patient 1 died 1 month post-SAbR from worsening heart failure owing to baseline severe stage D heart failure and severe aortic insufficiency; he had previously been on hospice for advanced heart failure and was referred for palliation of his recurrent ICD shocks. He had no pericardial or pleural effusion after therapy and had stable ejection fraction on echocardiogram. Autopsy did not show any unexpected myocardial necrosis outside of the known areas of targeted scar known previously from MRI, nor signs of coronary occlusion. Patient 4 died 3 months post-SAbR from respiratory failure owing to prior history of lung cancer and diffuse pulmonary fibrosis from amiodarone therapy. He did not experience pericardial or pleural effusion. The diffuse, hyperdense fibrosis pattern in the bilateral lower lobes (predominantly right lower lobe) seen on chest CT 17 cm away from the site of radiation was inconsistent with radiation pneumonitis and more suggestive of amiodarone-related pulmonary fibrosis.

#### Complications

**Figure 2:**
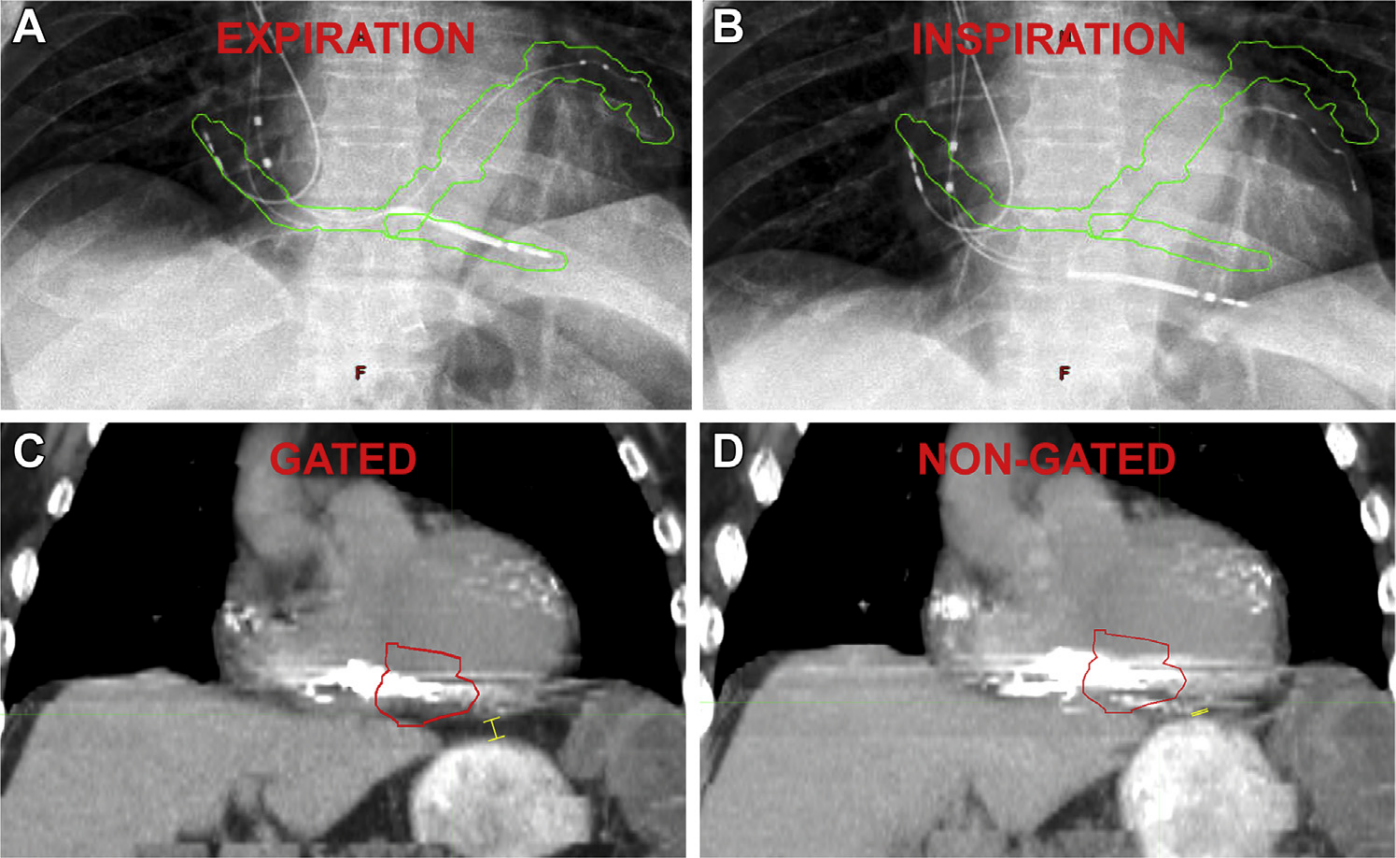
Respiratory-gated delivery of radiation. Example of patient 2, in whom respiratory-gated delivery was performed (see Supplemental Video 1). Fluoroscopy shows displacement of the implantable cardioverter-defibrillator (ICD) lead up to 2.2 cm during the respiratory cycle. The beam is turned on during expiration and off during inspiration, with the respiratory cycle tracked by an external optical tracking system. **A, B:** Proper timing of therapy is verified using intermittent fluoroscopy to confirm that the ICD lead is within the green contour during expiration (**A**) when beam is on and that the beam is off when the ICD lead is outside of the green contour during inspiration (**B**). **C:** On the gated expiratory phase 4-dimensional computed tomography study (4DCT), the inferior wall and ICD lead are clearly separated from the stomach (0.8 cm distance, *yellow marker*). **D:** On the nongated 4DCT, the inferior wall VT target (red planned treatment volume contour) and ICD lead are shown overlying the contrast-enhanced stomach (*yellow marker*).

Similar to prior reports, there was 1 case of pericardial effusion, which occurred 12 months after SAbR in patient 3, who presented with acute dyspnea. The serosanguinous effusion resolved after pericardiocentesis, and the drain was removed after 24 hours. There were no other acute or chronic toxicities from SAbR during follow-up. Notably, patients 2 and 5 had significant respiratory motion and VT targets (inferior basal RV+LV or inferior LV apex, Figure 2) in close proximity to the stomach and esophagus. Both had protocol-prescribed respiratory-gating and no study patients exhibited symptoms of GI side effects or gastropericardial fistula in median 6-month follow-up (12-month and 9-month follow-up in the 2 patients with inferior wall targets).

### Automated 12-lead ECG mapping results and comparison with manual analysis

**Figure 3:**
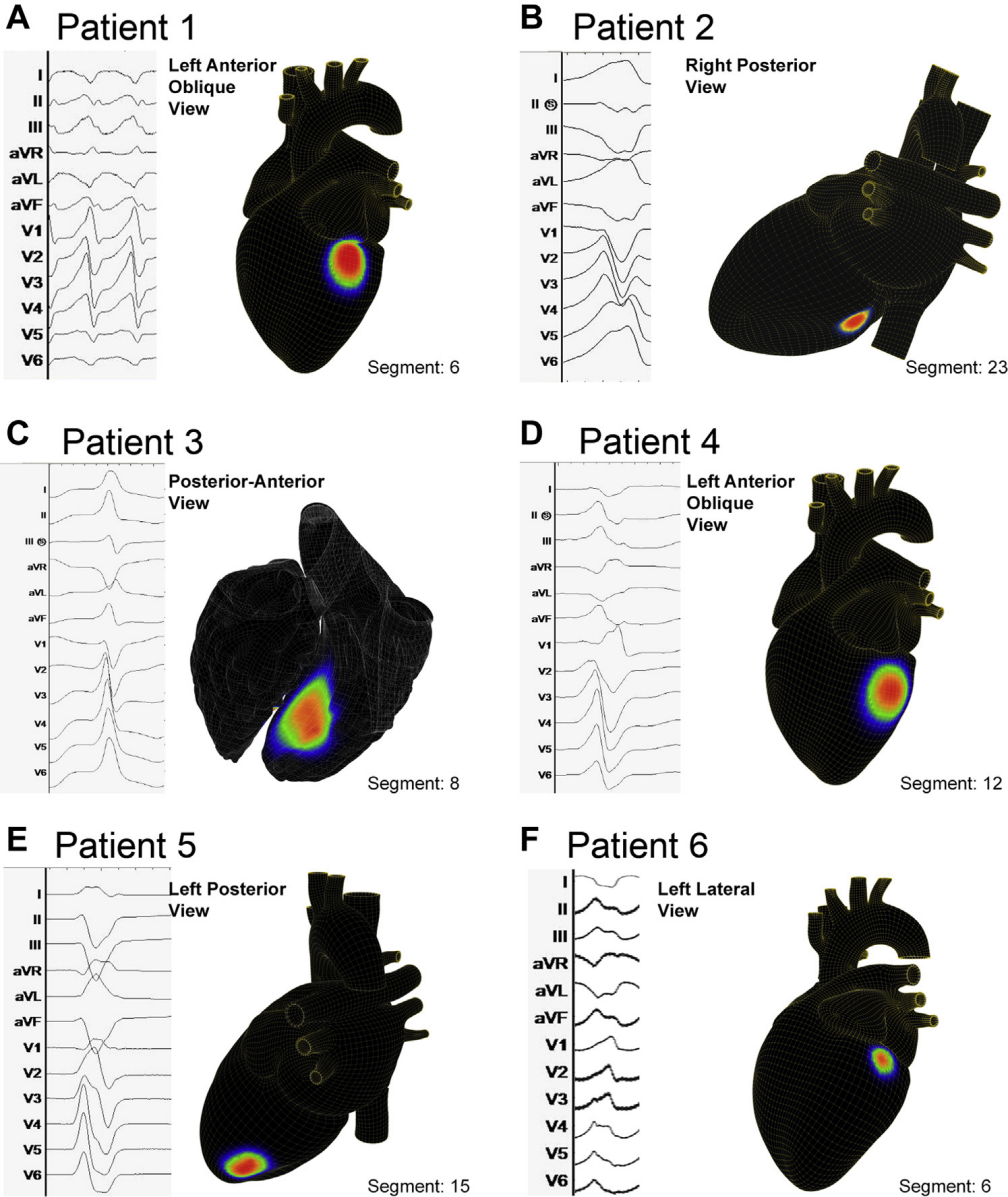
Noninvasive 12-lead electrocardiogram computational mapping. Example arrhythmia source mapping results and their corresponding cardiac segment are shown for this population with refractory ventricular tachycardia.

Noninvasive mapping using a computational ECG algorithm was performed in all patients, identifying 4.2 ± 2.3 VT morphologies per patient, with mean mapping time 1.1 ± 0.1 minutes per VT morphology. Figure 3 illustrates representative VT source locations for each patient localized by the automated ECG algorithm.

A total of 7 distinct VT morphologies in 3 patients had previously been mapped during invasive electrophysiology study; computational ECG mapping successfully localized the VT exit within the same cardiac segment identified using prior invasive activation mapping in all (7 of 7 VTs, 100%, Table 1), with further details in Supplemental Figure S4 and Supplemental Appendix, Section VI.

In comparison, the manual QRS morphology algorithm identified the correct segment of origin for 6 of 7 VTs (86%). The VT that was incorrectly localized by the manual QRS morphology algorithm originated from the inferior RV (Supplemental Figure S4).

### Respiratory cycle motion and CT wall thinning

Overall, the mean respiratory displacement (as measured by the ICD lead tip fiducial throughout the respiratory cycle) was 0.9 ± 0.7 cm with a maximum of 2.2 cm (patient 2). Figure 2 and Supplemental Video 1 show the ICD lead and CS leads in the anteroposterior (AP) view from this patient within a lead-tracking contour during end-expiration (A) and out of the contour during inspiration (B). Therapy delivery times were not significantly different between gated and nongated cases (12.1 ± 4.9 vs 8.4 ± 1.1 min, *P* = .3).

Notably, we found 3 of 4 of the nonischemic patients in this series exhibited areas of wall thinning localized by cardiac CT, which correlated with areas of low voltage or late gadolinium enhancement uptake on MRI when available. The patient who did not illustrate myocardial thinning at a VT exit site was study patient 3, who had an intramyocardial source in the interventricular septum, identified by electroanatomic mapping and the computational ECG algorithm; additional details are available in the Supplemental Appendix, section IV and Supplemental Figure S1.

### Gated and nongated SAbR treatment volumes

For patients in whom protocol-driven respiratory gating was used, the planning target volume (PTV) was smaller compared to patients who were not gated (71 ± 7 cc vs 153 ± 35 cc, p<0.01, Table 2).

**Table 2:** Cardiac and respiratory motion and clinical outcomes

	Patient 1	Patient 2	Patient 3	Patient 4	Patient 5	Patient 6
Cardiac cycle motion (cm)	0.29	0.58	0.55	0.42	0.36	0.35
Respiratory cycle motion (cm)	0.4	2.22	0.58	0.67	0.65	0.5
Respiratory gating	No	Yes	No	Yes	Yes	No
On-beam treatment time (min)	9.6	16.1	7.4	13.6	6.6	8.3
On-table treatment time	16.0	26.0	18.9	28.1	19.0	18.7
Delivered PTV (cc)	129	76	193	136	66	112
VT sources treated	6	3	2	1	5	7
Post-therapy ICD shocks	2	2	0	0	0	0
Complication	No	No	Pericardial effusion 12 months after therapy	No	No	No

ICD = implantable cardioverter-defibrillator; PTV = planned treatment volume; VT = ventricular tachycardia.

Analyzing the potential effects of respiratory-gated planning in all study patients, PTVs were significantly smaller when contoured using the expiration 4-dimensional computed tomography study (4DCT) average 40–60 (AVG 40–60) phases vs using free-breathing 4DCT (117 ± 47 cc vs 134 ± 44 cc, *P* = .045), resulting in a mean PTV reduction of 14% ± 12%. Figure 2 shows an example of an inferior wall target that is located within the VT target contour on the end-expiratory gated CT (C), while the inferior wall moves significantly beyond the VT contour overlying the stomach on the nongated CT (D).

Notably, the reduction in PTV significantly correlated with increasing degree of respiratory motion (R^2^ = 0.96, Supplemental Figure S5). Table 2 lists cardiac fiducial motion during the cardiac cycle and respiratory cycle, actual treatment PTV volumes, radiation delivery times, and clinical efficacy outcomes for study patients.

### Cardiac cycle motion

The mean ICD tip displacement during the cardiac cycle was 0.43 ± 0.13 cm (range 0.29–0.58 cm) in the AP view. The mean VT target displacement during the cardiac cycle was 0.36 ± 0.10 cm (range 0.26–0.51 cm) in the AP view. In aggregate, the maximal cardiac motion of intracardiac fiducials throughout the cardiac cycle was <0.6 cm in all patients when the effects of breathing were negated. Representative cardiac 4DCT images taken during systole and diastole are shown in Supplemental Figure S6. Additionally, detailed cardiac motion measurements in orthogonal views of the VT target and ICD and CS leads from each patient are listed in Supplemental Table S2.

### Histologic findings

**Figure 4:**
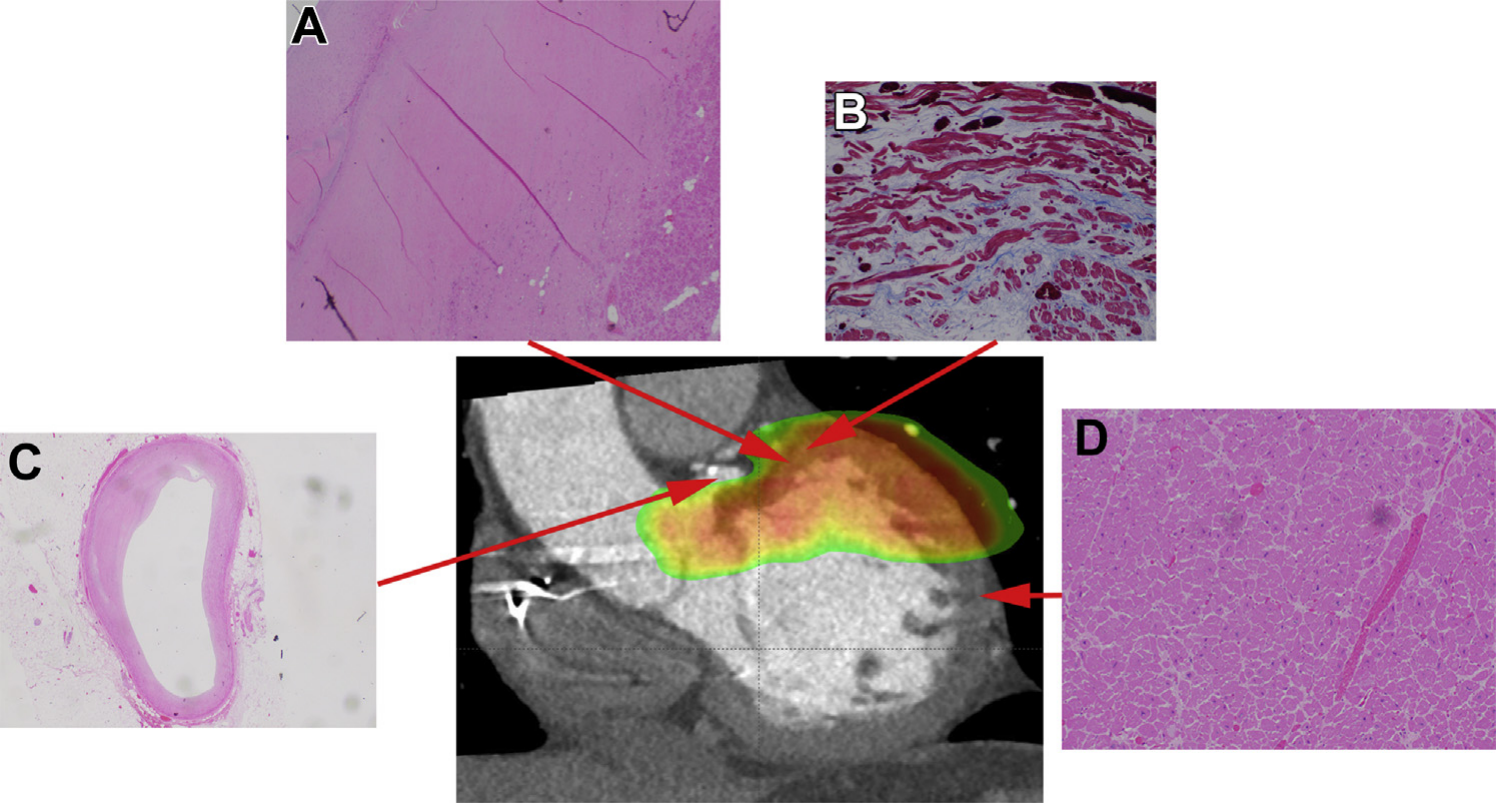
Histologic effects of stereotactic ablative radiotherapy for patient 1. **A:** Dense fibrosis at the basal anteroseptum. **B:** “Wavy fibers” mixed with fibrosis on trichrome stain of the basal anterior mitral annulus within the targeted region. **C:** No obstruction seen in the left circumflex coronary artery that was located in the targeted region. **D:** Preserved myocardial structure outside targeted areas.

Histologic examination from autopsy of patient 1 is shown in Figure 4; dense fibrosis and abnormal “wavy fibers” were seen in targeted regions (Figure 4A and 4B), consistent with radiation-induced myocardial injury, but not in untargeted regions.

### Proximity of the stomach to the heart

**Figure 5:**
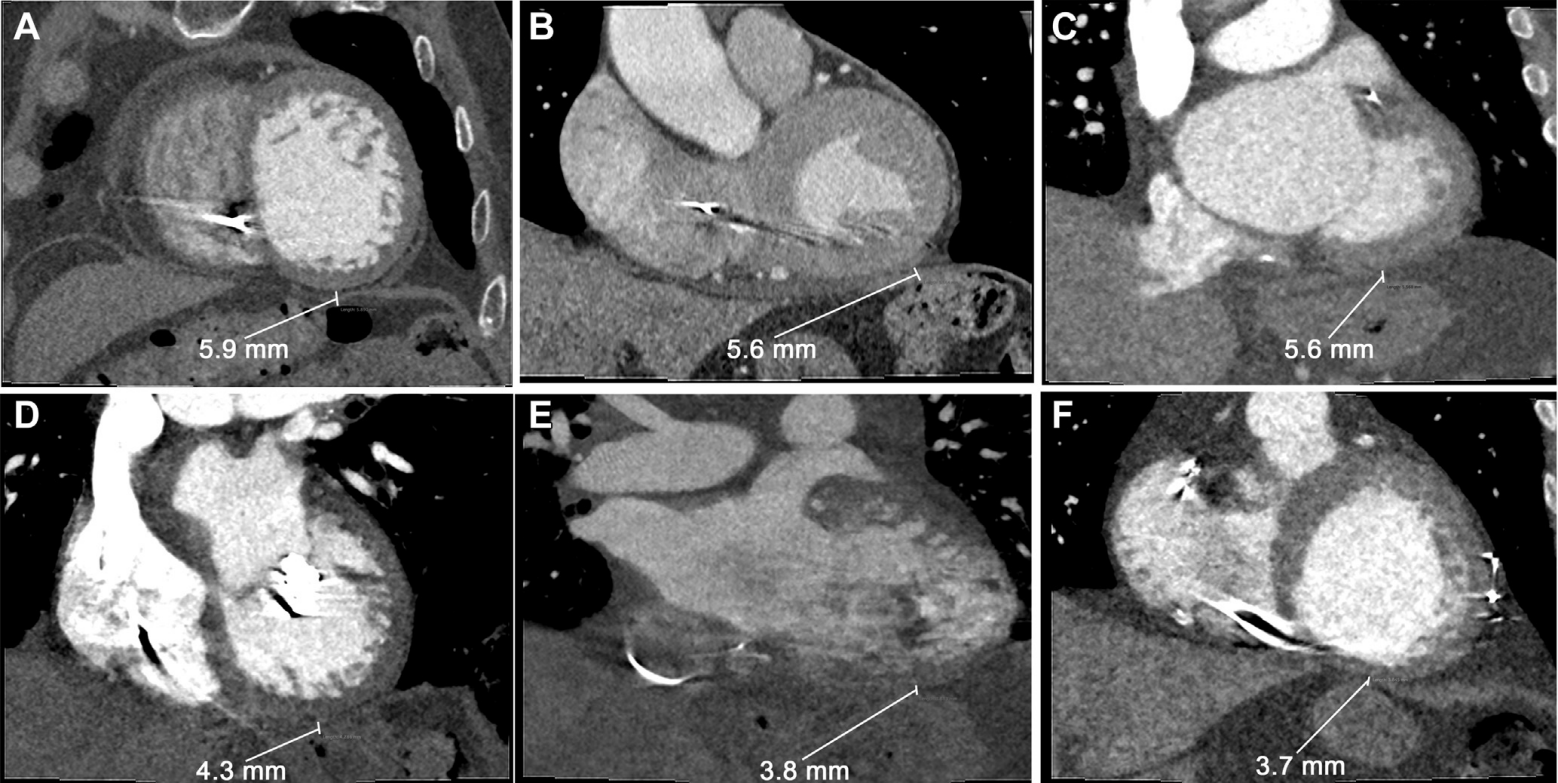
Close proximity of the inferior wall to the stomach. Computed tomography images from all patients showing the close proximity of the stomach to the basal inferior left ventricle wall (4.8 ± 1.0 mm) for each study patient. The smallest distance was 3.7 mm in patient 6.

The mean distance from the inferior LV wall to the stomach in all patients was 4.8 ± 1.0 mm with a minimum distance of 3.7 mm. Representative cardiac CT images visualizing the closest proximity of the heart to a GI structure in all patients are shown in Figure 5. The closest region of the ventricle to the GI system tended to be the basal inferior LV wall. The proximity of the stomach to the VT target in patients 2 and 5 were 9.0 mm and 3.8 mm, respectively.

## Discussion

This pilot study highlights 3 key findings that may improve the planning and delivery of cardiac radioablation. First, computational 12-lead ECG mapping is feasible and may facilitate target planning via 3-D visualization of VT exit sites with precision appropriate for noninvasive VT ablation. Second, respiratory motion may vary widely between patients, and protocol-guided respiratory-gated delivery of radioablation appears feasible and may improve treatment precision in patients with significant respiratory motion and targets in close proximity to the stomach. Third, the inferior LV is a high-risk region for SAbR therapy owing to its proximity to gastroesophageal structures, providing additional rationale for respiratory gating in patients with VT sources in this region.

### Automated ECG mapping to facilitate VT targeting

Advantages of computational-based ECG analysis include visualization of algorithm results on a 3-D model to facilitate target planning in the noninvasive workflow. Although the present study population is small, the agreement between the computational ECG algorithm output and the results from prior electroanatomic mapping provides support for the utility of this tool for patients with advanced structural heart disease seen in this population. Additionally, 12-lead ECG mapping is feasible for patients with heart failure and generally well tolerated.

In some centers, manual interpretation of VT QRS morphology has been used to help guide SAbR.^6^^,^^9^^,^^10^ Potential limitations to this approach include a suboptimal resolution of targets originating from the right ventricle, as seen for 1 VT in this case series. Contemporary studies have also shown limited accuracy of manual QRS morphology analysis (range 39%–82%) compared with invasive mapping.^17^

In prior work, ECGi systems have been used to map VT exit sites.^7^ Unlike the ECGi workflow, which requires use of a mapping vest and concurrent CT scan, the proposed SAbR workflow requires only the digital 12-lead ECG data of the ventricular arrhythmia and proprietary software to map VT targets, potentially enhancing access to SAbR therapy.

### Respiratory-gated radioablation feasibility

Respiratory motion compensation is commonly used by electroanatomic mapping systems during invasive catheter ablation procedures to minimize catheter movement artifacts, improving accuracy and contact force.^18^, ^19^, ^20^ It is also used routinely for stereotactic body radiotherapy to treat intrathoracic tumors such as lung cancer and was shown to decrease PTV size and toxicity to normal tissue.^18^ Presently, however, respiratory gating is uncommonly used in cardiac SAbR; abdominal binders or no mitigation of respiratory motion have been the most common approaches.

In this study we evaluated the feasibility of a protocol-driven gating protocol, with the goal of performing respiratory gating on patients most likely to benefit. We found that this protocol was feasible and safe, and resulted in approximately half of patients undergoing gated procedures. Although it was a small study, no GI complications were noted in patients with inferior LV VT targets.

### Respiratory gating increases PTV precision

We found that respiratory-gated PTVs were significantly smaller than nongated PTVs despite a similar number of arrhythmia targets. Our data suggest that respiratory gating may increase precision by reducing PTV volume in proportion to respiratory cardiac motion.

The optimal cutoffs in which to perform respiratory gating had previously been unclear. We measured the maximal displacement of all fiducials in orthogonal views (including ICD and CS lead tips) during cardiac contractile motion to be <0.6 cm. This is consistent with prior studies assessing motion from cardiac contraction during the cardiac cycle, and supports using a respiratory displacement threshold of ≥0.6 cm, since motion from the cardiac cycle is not accounted for with respiratory gating.^15^ Further studies are needed to determine whether there is a need to additionally gate for cardiac cycle motion to further improve precision below 0.6 cm.

In previous reports, a vacuum-assisted body immobilizer was used to limit respiratory motion.^4^^,^^7^ We developed the present workflow to increase patient comfort and address our concern that our patients with advanced heart failure and orthopnea may not tolerate a body immobilizer. We noted a maximum cardiac excursion during respiration of 2.2 cm, supporting the cutoff of a VT target within 2.0 cm of a GI structure. Further studies are required to determine whether different cutoff values provide enhanced safety and efficacy.

Notably, our protocol for respiratory gating does not involve placement of a transvenous pacing wire as a radiographic fiducial, which is required for some linear accelerators utilizing fiducial tracking systems unable to track existing transvenous ICD systems. The proposed workflow uses fluoroscopy of existing intracardiac fiducials (ICD and CS lead tips) to confirm accurate gating. Our respiratory gating strategy does not rely entirely on tracking the motion of the fiducial as a surrogate of the target, but the fiducials are contoured to confirm alignment of the heart through respiration.

### Safety of inferior wall arrhythmia targets

Prior work has demonstrated potential complications of radiation delivery adjacent to GI structures such as gastropericardial fistula.^21^^,^^22^ Our study reveals that the inferior ventricular wall is a particularly high-risk region. We found that the stomach can be less than 4 mm away from the VT target while respiratory motion can be up to 22 mm. Notably, respiratory-gated radiation delivery may be an important method to mitigate risk and minimize potential collateral damage of GI and pulmonary structures. While small, our series included respiratory-gated delivery in 2 patients with inferior wall targets near the stomach. No complications were noted in these patients at 12- and 9-month follow-up with effective arrhythmia suppression.

### Histology of radioablation

Despite successful radioablation with significant ICD shock reduction, study patient 1 experienced progression of his end-stage heart failure owing to severe aortic insufficiency at 1 month post-SAbR. At autopsy, targeted regions displayed mixed dense and patchy fibrosis, with preserved myocardial cells in nontargeted regions, similar to a prior case report.^7^ Additionally, we observed the presence of “wavy fibers” intermixed with fibrosis within the targeted region, likely reflecting the effects of cardiac radiation, which ultimately results in fibrosis.^23^ Additional studies are needed to evaluate the precise long-term effects of SAbR therapy on myocardial tissue.

### Limitations

First, the study is limited by small sample size; however, the workflow feasibility, efficacy, and procedural safety may be of interest to centers looking to begin SAbR therapy who do not have access to ECGi. Second, while we do compare the automated ECG mapping results to a manual QRS morphology algorithm, we do not directly compare mapping results with ECGi; future studies are required to directly compare workflow differences and mapping accuracy between these 2 systems. Third, while the criteria of respiratory motion ≥2.0 cm and target proximity ≤0.6 cm were based on our study population data, larger studies are required to validate these cutoffs as metrics to guide respiratory gating. Fourth, we evaluated mapping accuracy of the computational 12-lead ECG mapping system based upon identification of the correct segment of the cardiac model. While this is state of the art for SAbR planning, additional work is required to provide a more precise spatial estimate of mapping accuracy. Fifth, the precise mapping algorithms are proprietary, although the robust agreement with invasive electroanatomic mapping supports utility of this approach in this population. Future validation studies in larger populations are in progress. Sixth, use of CT wall thinning analysis to identify arrhythmogenic substrate has been validated in ischemic cardiomyopathy, but not yet in nonischemic cardiomyopathy. In our series, wall thinning was observed in proximity VT exit sites in 75% of patients with nonischemic cardiomyopathy patients, supporting it use in this context. Further studies evaluating CT in nonischemic cardiomyopathy are in progress. Seventh, the study is limited by short follow-up, given that the gastropericardial fistula has been reported more than a year after treatment. Nevertheless, the 2 patients with inferior wall targets were followed out to 1 year (patient 2) and 9 months (patient 5) without any GI system side effects or toxicities. Finally, although it is unknown whether the ICD lead tip may directly reflect the cardiac motion of a VT target, respiratory gating does not entirely depend on active tracking of a fiducial to direct the beam; our data suggest that the motion of the cardiac cycle appears small (<6 mm) while respiratory motion may range up to 22 mm, and the chosen fiducial is only used if its motion moves with the VT target, as seen on planning respiratory 4DCT. Future studies are needed to directly track motion of the VT target for increased precision.

## Conclusion

Noninvasive computational ECG mapping and protocol-based respiratory gating may help facilitate the radioablation planning workflow and provide short-term safety and maintain efficacy during SAbR therapy in patients with advanced structural heart disease and refractory VT. Notably, the basal inferior LV is a particularly high-risk region for SAbR for GI involvement, with average separation of critical sites below the threshold of cardiac motion. Larger studies with longer follow-up are in progress to further validate these findings.
