# Supplementary material for: Computational ECG mapping and respiratory gating to optimize stereotactic ablative radiotherapy workflow for refractory ventricular tachycardia
Source: Heart Rhythm O2. 2021 Sep 20;2(5):511–20. doi: 10.1016/j.hroo.2021.09.001 (PMC8505208; doi:10.1016/j.hroo.2021.09.001)
Supplement: Supplemental Appendix [file mmc1.docx]

**Online Supplemental Appendix**

Gordon Ho, MD, FHRS^1^

Todd F. Atwood, PhD^2^

Andrew R. Bruggeman, MD^2^

Kevin L. Moore, PhD^2^

Elliot McVeigh, PhD^3^

Christopher T. Villongco, PhD^4^

Frederick T. Han, MD, FHRS^1^

Jonathan C. Hsu, MD, MAS, FHRS^1^

Kurt S. Hoffmayer, MD, PharmD, FHRS^1^

Farshad Raissi, MD, FHRS^1^

Grace Y. Lin, MD, PhD^5^

Amir Schricker, MD, MS, FHRS^6^

Christopher E. Woods, MD, FHRS^6^

Joey P. Cheung, PhD^7^

Al V. Taira, MD^7^

Andrew McCulloch, PhD^3^

Ulrika Birgersdotter-Green, MD, FHRS^1^

Gregory K. Feld, MD, FHRS^1^

Arno J Mundt, MD^2^

David E. Krummen, MD, FHRS^1^

^1^Department of Medicine-Cardiology, UCSD, La Jolla, CA

^2^Department of Radiation Medicine, UCSD, La Jolla, CA

^3^Department of Bioengineering, UCSD, La Jolla, CA

^4^Vektor Medical Inc, Carlsbad, CA

^5^Department of Pathology, UCSD, La Jolla, CA

^6^Department of Cardiac Electrophysiology and ^7^Department of Radiation Oncology, Mills-Peninsula Medical Center, Sutter Health, Burlingame, CA

Running Title: *ECG Mapping and Respiratory-Gated VT Radiotherapy*

Correspondence to:

Gordon Ho, MD, FACC, FHRS

3350 La Jolla Village Drive

Cardiology Section 111A

San Diego, CA 92161

Email: goho@health.ucsd.edu

**Supplemental Index: Table of Contents**

1. Methods: Non-invasive VT Induction and Computaional ECG Mapping
2. Methods: Detailed Respiratory Gating Protocol
3. Methods: Retrospective Respiratory and Cardiac Cycle Motion Analysis
4. Methods/Results: Use of Cardiac CT-derived Wall Thinning to Localize Diseased Substrate
   1. Figure S1: Wall thinning in non-ischemic cardiomyopathy patient (Patient #1)
   2. Figure S2: Limited cardiac MRI in non-ischemic cardiomyopathy patient correlating to wall thinning near VT exit sites (Patient #1)
5. Methods/Results: Automated ECG Mapping Identifies Multiple VT Exit Sites Correlating with Wall Thinning
   1. Figure S3. Example of a Patient with Prior Inferior Infarct
6. Methods/Results: Comparison of Automated Computational ECG Mapping Against Manual Visual ECG Interpretation
   1. Figure S4: Example Comparison Between Manual QRS Morphology Algorithm and Computational ECG Algorithm
7. Results: Detailed Patient Demographics
   1. Table S1: Additional clinical characteristics of the study population
8. Results: Correlation of PTV reduction with degree of respiratory motion
   1. Figure S5: Relationship between magnitude of fiducial displacement during respiratory motion and the associated reduction in planned treatment volume
9. Results: Limited Cardiac Displacement during Ventricular Systole Measured Using Fluoroscopic Tracking of Intracardiac Fiducials
   1. Figure S6: Limited cardiac motion throughout the cardiac cycle
   2. Table S2: Motion during the Cardiac Cycle of the VT Target and Intracardiac Leads in Orthogonal Views from 4-Dimensional Cardiac Computed Tomography
10. Results: Duration of Planning and Treatment Times
11. Results: ATP Reduction
12. **Supplemental Methods: Non-invasive VT Induction and Computational ECG Mapping**

Patients underwent non-invasive programmed stimulation (NIPS) using their Implantable Cardioverter-Defibrillator (ICD) to perform ventricular extra-stimulus or rapid pacing for VT induction (Cuculich, NEJM 2018) in the electrophysiology laboratory. Pacing consisted of delivering single or double extra stimuli pacing following a standard 8 beat drive train in the electrophysiology laboratory. The pacing protocol was repeated to initiate different VT morphologies until a recurrent VT morphology, defined as a VT morphology on 12 lead ECG that was identical in 12 of 12 ECG leads to a previously induced VT episode, was recorded. The induction protocol was intended to elicit the spectrum of clinical VTs observed on ICD interrogation or on ECGs in the hospital. The 12-lead ECGs of VT were recorded using an electrophysiology recording system (either Bard Pro, Boston Scientific, Marlborough, MA or Workmate Claris System, St. Jude, Saint Paul, MN). The 12 lead ECG data can be recorded using standard typical filtering settings (0.05 – 150 Hz) from any standard digital recording system available in electrophysiology laboratories (BARD, Prucka CardioLab, Workmate Claris) or portable digital 12-lead ECG recording systems that can be used in the outpatient or emergency room setting (Nasiff CardioCard).

The computational ECG mapping system was developed in the Python programming language (Python Software Foundation, Delaware, USA) to perform non-invasive, beat-by-beat, multi-chamber mapping of the heart. It incorporates a strategy whereby ECG data from the patient is compared to a large pre-computed library of arrhythmia simulations (>1 million arrhythmia-cycles). The arrhythmia library is based on finite element models of cardiac electrophysiology and simulated using the Continuity problem solving environment developed by the Cardiac Mechanics Research Group at the University of California San Diego.^1–3^

The software requires a digitized 12-lead ECG of the clinical arrhythmia. Additionally, patient-specific parameters such as the location of large scar and the presence of ventricular dilation or hypertrophy can be entered into the algorithm, if present clinically.

Algorithm results are used to compose 3-dimensional heatmaps visually representing the arrhythmia source probability analysis, as noted in manuscript Figure 3. These results were then manually transferred to the Varian Eclipse Treatment Planning Software (Aria Version 16.00.00, Varian Medical Systems Inc, Palo Alto, CA) for ablation planning.

1. **Methods: Detailed Respiratory Gating Protocol**

Prior to radiation delivery, potential respiratory-gating fiducials were assessed as to their degree of cardiac motion through the respiratory cycle as measured from a free-breathing 4-dimensional computed tomography study (4DCT) and their proximity to the target site. Potential fiducials included the ICD lead, coronary sinus lead, Mitra-clip, and left ventricular assist device. Radiation simulation end-expiration CTs and respiratory 4DCTs (10 phases) were performed, and the degree of respiratory displacement was measured from the fiducials (ICD and/or CS lead tips) and cardiac structures to determine the need for respiratory gating based on the SAbR gating assessment protocol (Graphical Abstract, middle left panel). As described above, if there was significant respiratory displacement (≥0.6cm) or the close proximity of the VT target to the esophagus or stomach (≤2.0 cm) during fluoroscopy, the VT target volumes were contoured on an average image of the end expiration 4DCT phases (AVG40-60 CT). Then a control volume encompassing the lead fiducials were contoured onto the same AVG40-60 CT (Graphical Abstract , middle right panel). If there was no significant respiratory displacement, then the VT target volume and fiducials were contoured on an average image of all 4DCT phases (AVG0-90 CT) to treat the target during the patient’s entire breathing cycle.

1. **Methods: Retrospective Respiratory and Cardiac Cycle Motion Analysis**

To assess the impact of respiratory gating on therapy volume, internal target volumes (ITVs) and planning target volumes (PTVs) for both the expiratory phases (AVG40-60 CT) and all phases of the respiratory cycle (AVG0-90 CT) were calculated for all patients and compared.

The degree of cardiac cycle motion was analyzed using 4D cardiac CT (details in Supplement Section IV).

1. **Methods/Results: Use of Cardiac CT-derived Wall Thinning to Localize Diseased Substrate**

The correlation of CT-derived wall thinning <5 mm with diseased arrhythmogenic substrate has been previously validated for infarct-related cardiomyopathy in the literature by independent groups.^4–7^ We found that the use of cardiac CT to identify diseased substrate was particularly helpful in our workflow for several reasons. First, use of the CT for substrate characterization significantly simplified the radiotherapy workflow because all target contouring is eventually performed on a CT scan within the radioablation treatment planning system. CT substrate analysis reduced the additional potential registration errors of substrate locations from other modalities to the planning CT. Second, CT wall thinning was used for substrate characterization because of our institutional expertise in this field.^8^ Lastly, current generation cardiac CT has greater spatial resolution (0.5x0.5x0.5mm for cardiac CT compared to 1.5x1.5x6mm for MRI).^9^

Overall in our series, wall thinning using cardiac CT analysis was identified near the VT exits in most patients (5 out of 6 patients, 83%). MRI was available only in 2 patients for various reasons, such as contraindications to MRI due to claustrophobia (Patient #1), significant artifact from Mitra-clips near the region of interest (Patient #4) and presence of an LVAD (Patient #6).

Although the ability of wall thinning to identify arrhythmogenic substrate has not yet been specifically studied in the non-ischemic cardiomyopathy population, we found 3 of 4 of the non-ischemic patients in this series exhibited areas of wall thinning localized by cardiac CT, which correlated with areas of low voltage or LGE uptake on MRI when available. The patient who did not illustrate myocardial thinning at a VT exit site was study patient 3, who had a intramyocardial source in the interventricular septum, identified by invasive electroanatomic mapping and the computational ECG algorithm.

Figure S1 illustrates an example of CT wall thinning at the VT target for Patient #1. In this region, LV wall thickness ranged from 1.7 to 3.9 mm, below the 5mm threshold for scar. Figure S1 part A shows a left anterior oblique image, while B shows a long axis view of the left ventricle. Figure S2 demonstrates LGE uptake on the MRI (red arrow) correlating with wall thinning seen on CT extending from the septum to the basal anteroseptum.

**Figure S1:** Wall thinning in non-ischemic cardiomyopathy patient (Patient #1)


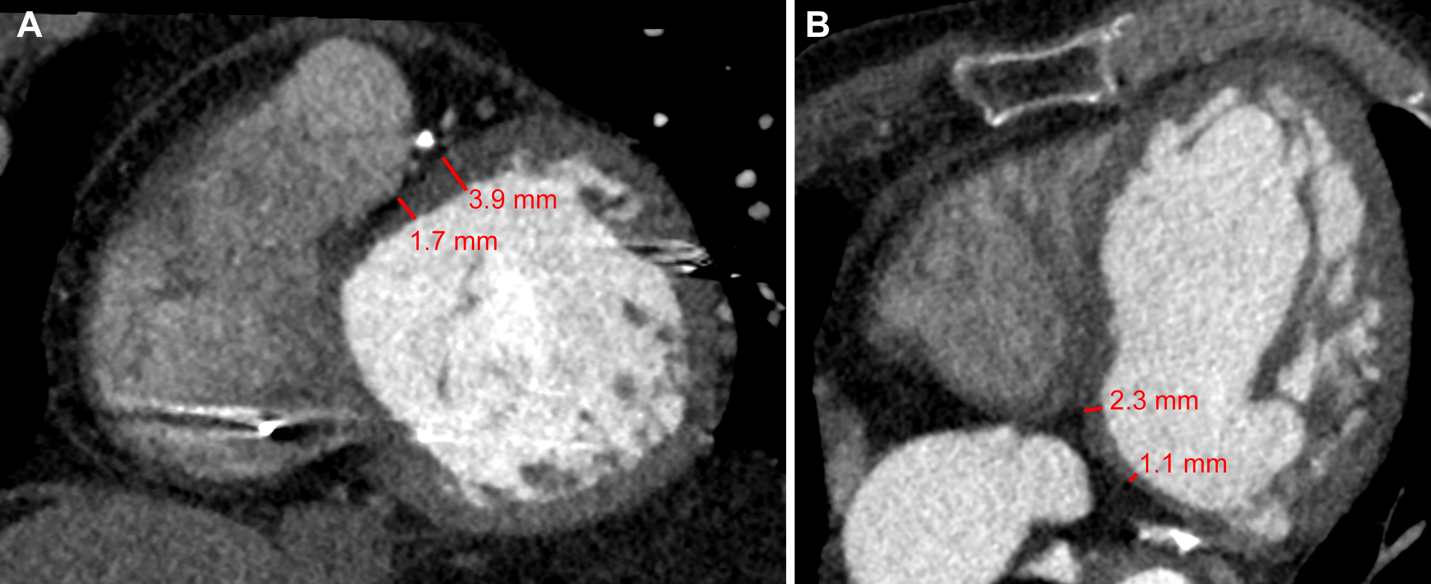


**Figure S2:** Limited cardiac MRI in non-ischemic cardiomyopathy patient correlating to wall thinning near VT exit sites (Patient #1)

*
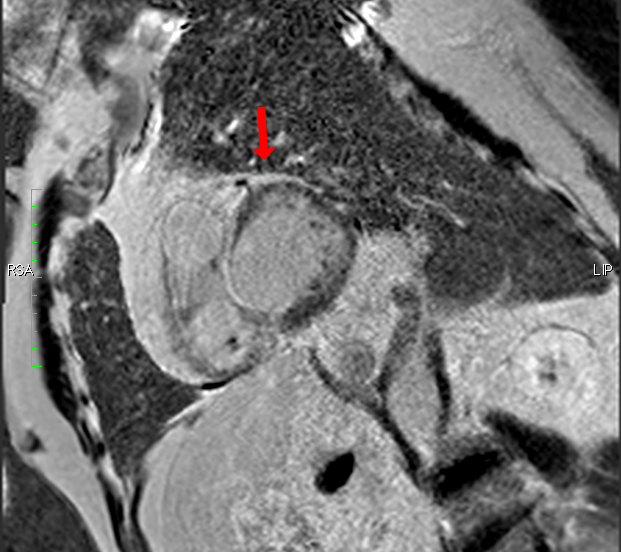
*

A second example of regional wall thinning at a VT exit site is shown in Figure S3 (Section V), which shows an example of a patient with a prior inferior MI (Patient 5) with multiple VT exit sites (A and B, left and middle panels) exiting around a large inferoapical scar, correlating with wall thinning localized from cardiac CT (A and B, right panels).

1. **Methods/Results: Automated ECG Mapping Identifies Multiple VT Exit Sites Correlated with Wall Thinning**

**Figure S3.** Example of a Patient with Prior Inferior Infarct with 2 VT Morphologies Correlating with Wall Thinning Identified from Cardiac CT


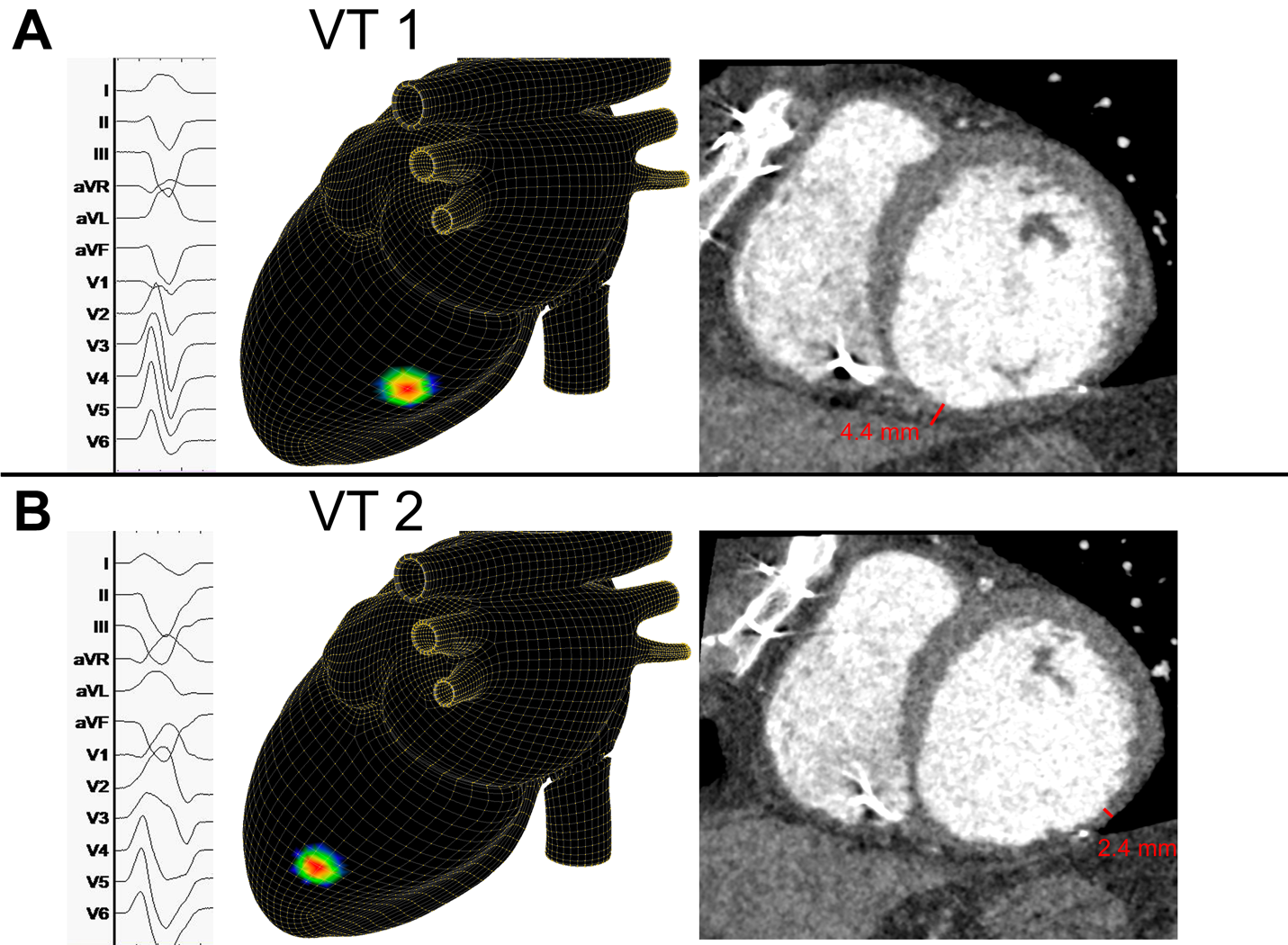


1. **Methods/Results: Comparison of Automated Computational ECG Mapping Against Manual Visual ECG Interpretation**

We compared the mapping results for the automated ECG algorithm versus the manual VT QRS morphology algorithm for 7 distinct VT for which electroanatomic mappng was available as a gold-standard. We found that the automated ECG algorithm correctly identified the correct segment in 7 of 7 VTs, while the manual QRS morphology algorithm correctly identified 6 of 7 VTs (86%). The VT incorrectly localized by the manual QRS morphology algorithm had a site of origin within the inferior right ventricle. The accuracy of the manual algorithm was reported to be 82% in the initial study by Andreu and colleagues 2018, but a subsequent study showed that the accuracy was as low as 39% to the correct cardiac segment when compared to invasive mapping.^10,11^ Figure S4 demonstrates an example of a VT (far left panel) originating from the basal inferior RV by epicardial activation mapping (far right panel). This was correctly localized by novel computational ECG mapping (middle right panel), but incorrectly localized by the manual ECG algorithm (middle left panel).

**Figure S4 Example Comparison Between Manual QRS Morphology Algorithm and Computational ECG Algorithm**

**
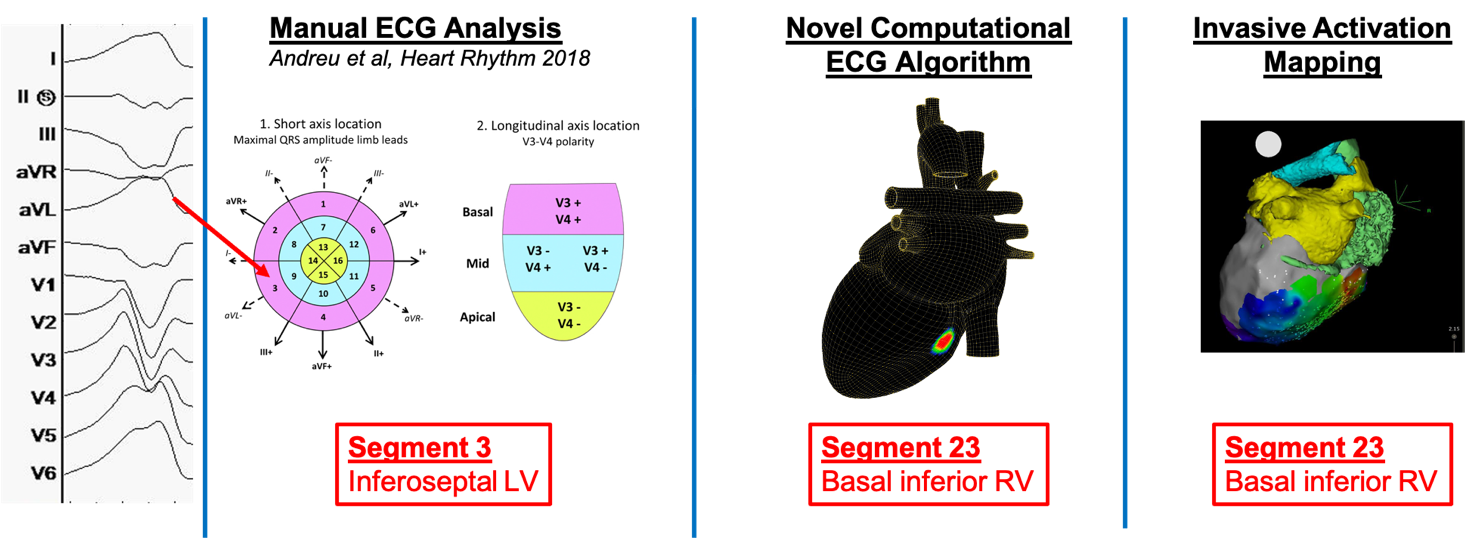
**

1. **Results: Detailed Patient Demographics**

**Table S1:** Additional clinical characteristics of the study population are shown. Data are representative of a study population with severe comorbidities.

| **Subject #** | **Patient 1** | **Patient 2** | **Patient 3** | **Patient 4** | **Patient 5** | **Patient 6** |
| --- | --- | --- | --- | --- | --- | --- |
| **Indication** | VT storm | VT storm | VT storm | Refractory VT | VT storm | VT storm |
| **Patient Status at Referral** | Inpatient | Inpatient | Inpatient | Inpatient | Outpatient | Inpatient |
| **Contraindication to Invasive Ablation** | Intracardiac aortic valve mass, severe aortic regurgitation, frailty, failed VT ablations | Epicardial VT source at PDA artery, failed VT ablations | Intramural septal VT, failed ablations including bipolar | Acute subdural hemorrhage, frailty, failed VT ablation | Intracardiac LV thrombus in LV aneurysm, | Epicardial LV summit at the left main artery, prior sternotomies from CABG and LVAD, failed VT ablations |
| **Relevant Comorbidities** | Stage D heart failure, severe aortic regurgitation, CKD IV, previously on hospice | CAD s/p PCI, Stage C heart failure | Stage C heart failure | Stage D heart failure, severe mitral regurgitation s/p Mitra-Clip, h/o lung cancer s/p resection, dysphagia with recurrent aspiration pneumonitis, previously on hospice | Stage D heart failure, CAD s/p CABG and PCI | Stage D heart failure, LVAD |

**Key:** VT: ventricular tachycardia, PDA: posterior descending artery, LV: left ventricle, CABG: coronary artery bypass grafting, LVAD: left-ventricular assist device, CKD: chronic kidney disease, CAD: coronary artery disease, PCI: percutaneous coronary intervention

1. **Results: Correlation of PTV reduction with degree of respiratory motion**

A linear correlation plot was created to correlate the percent reduction of respiratory-gated PTV versus the respiratory displacement.

**Figure S5:** Relationship between magnitude of fiducial displacement during respiratory motion and the associated reduction in planned treatment volume for stereotactic ablative radiotherapy.


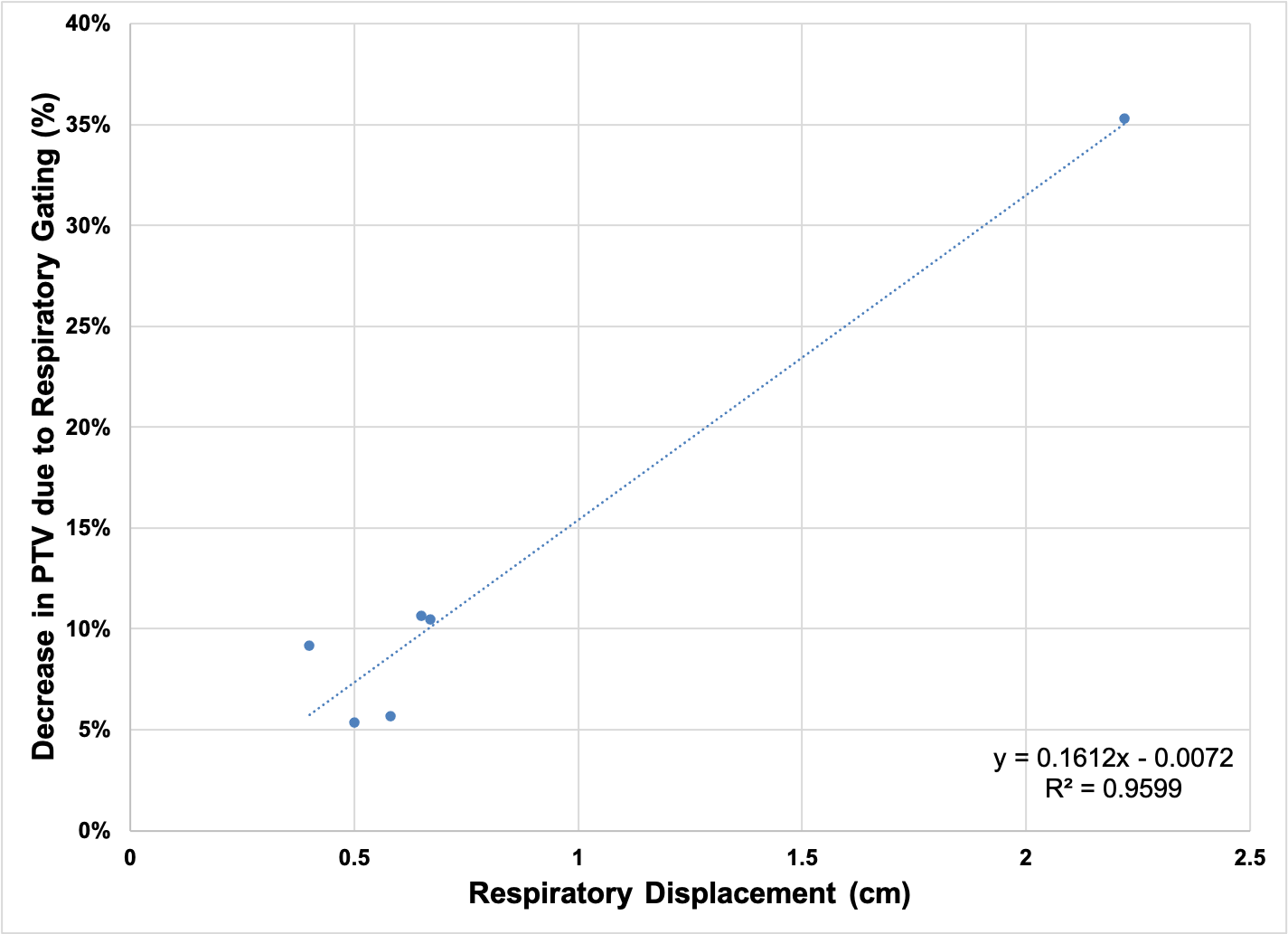


1. **Results: Cardiac Displacement during Ventricular Systole Measured Using Fluoroscopic Tracking of Intracardiac Fiducials**

Multiple potential fiducial markers (CS lead, LVAD, Mitra-clip) were considered for use as fiducials. Because intracardiac markers may not share the same cardiac motion trajectories as the VT target, an attempt was made to characterize cardiac cycle motion of the RV lead, CS lead, and VT target individually (Fig S2 and Table S2).

To determine the degree of cardiac motion of intracardiac fiducials through the cardiac cycle, the displacement of multiple radiographic fiducials was measured in orthogonal views on a maximal-intensity projection (MIP) cardiac-gated 4DCT reconstruction (Horos, Horos Project, Annapolis, MD, USA).

**Figure S6.** Limited cardiac motion throughout the cardiac cycle in a patient (Pt #1) with reduced EF of 23% is shown. (A) Cardiac contraction during systole, (B) diastole and (C) overlying images of systole and diastole showing displacment of the endocardial wall, ICD and CS lead fiducials.


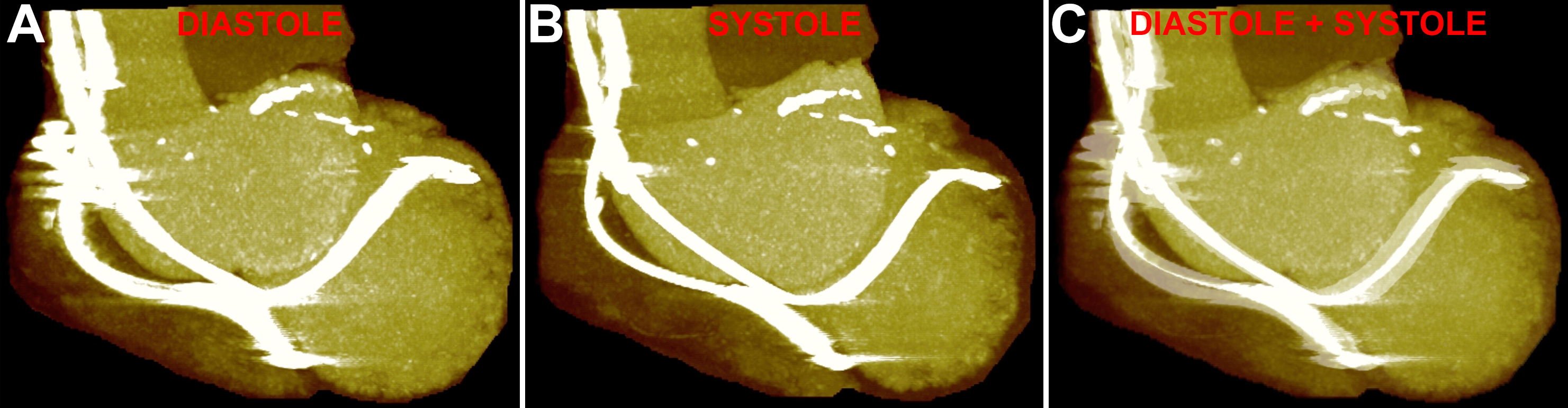


**Table S2.** Motion during the Cardiac Cycle of the VT Target and Intracardiac Leads in Orthogonal Views from 4-Dimensional Cardiac Computed Tomography

| **Subject** | **Target (AP) cm** | **Target (LL)** | **ICD (AP)** | **ICD (LL)** | **CS (AP)** | **CS (LL)** |
| --- | --- | --- | --- | --- | --- | --- |
| 1 | 0.26 | 0.3 | 0.29 | 0.1 | 0.5 | 0.4 |
| 2 | 0.4 | 0.55 | 0.58 | 0.58 | NA | NA |
| 3 | 0.33 | 0.28 | 0.55 | 0.3 | 0.53 | 0.29 |
| 4 | 0.51 | 0.36 | 0.42 | 0.39 | NA | NA |
| 5 | 0.38 | 0.25 | 0.36 | 0.26 | 0.22 | 0.28 |
| 6 | 0.26 | 0.2 | 0.35 | 0.2 | 0.21 | 0.19 |

**Key:** AP: antero-posterior view, ICD: implantable cardioverter-defibrillator, CS: coronary sinus, LL: left lateral

1. Results: Duration of Planning and Treatment Times
2. Time from Referral to Treatment:

The average time from patient selection (referral for non-invasive radioablation) to treatment delivery was 21±2 days

1. On-beam Treatment Time: (Refer to Table 2 in main manuscript for individual treatment times)

The mean on-beam treatment time for respiratory-gated patients was 12.1±4.9 minutes compared to non-gated patients was 8.4±1.1 minutes (p=0.3).

1. On-table Treatment Time: (Refer to Table 2 in main manuscript for individual treatment times)

The mean total on-table times for respiratory gated patients were 24.4±4.8 min compared to non-gated patients were 17.8±1.6 (p=0.08).

1. **Results: ATP Reduction**

ATP events decreased from 898 ATPs in the 30 patient-months prior to therapy to 72 ATPs in the 27 patient-months after therapy (relative reduction 92%). Patient #6 did not have ATP data available because it was subsequently turned off 1 month prior to SAbR due to incessant VT storm resulting in sedation/intubation with termination of significant VT limited to external cardioversion, but was turned back on after SAbR therapy.

**References**

1. Villongco, C. T., Krummen, D. E., Stark, P., Omens, J. H. & McCulloch, A. D. Patient-specific modeling of ventricular activation pattern using surface ECG-derived vectorcardiogram in bundle branch block. *Prog Biophys Mol Biol* **115**, 305–313 (2014).

2. Vincent, K. P. *et al.* High-order finite element methods for cardiac monodomain simulations. *Front Physiol* **6**, 217 (2015).

3. Krishnamurthy, A. *et al.* Patient-Specific Models of Cardiac Biomechanics. *J Comput Phys* **244**, 4–21 (2013).

4. Takigawa, M. *et al.* Are wall thickness channels defined by computed tomography predictive of isthmuses of postinfarction ventricular tachycardia? *Heart Rhythm* **16**, 1661–1668 (2019).

5. Tian, J. *et al.* Three-dimensional contrast-enhanced multidetector CT for anatomic, dynamic, and perfusion characterization of abnormal myocardium to guide ventricular tachycardia ablations. *Circulation: Arrhythmia and Electrophysiology* **3**, 496–504 (2010).

6. Cochet, H. *et al.* Integration of merged delayed-enhanced magnetic resonance imaging and multidetector computed tomography for the guidance of ventricular tachycardia ablation: A pilot study. *Journal of Cardiovascular Electrophysiology* **24**, 419–426 (2013).

7. Komatsu, Y. *et al.* Regional myocardial wall thinning at multidetector computed tomography correlates to arrhythmogenic substrate in postinfarction ventricular tachycardia: Assessment of structural and electrical substrate. *Circulation: Arrhythmia and Electrophysiology* **6**, 342–350 (2013).

8. Behar, J. M. *et al.* Comprehensive use of cardiac computed tomography to guide left ventricular lead placement in cardiac resynchronization therapy. *Heart Rhythm* **14**, 1364–1372 (2017).

9. Mahida, S. *et al.* Cardiac imaging in patients with ventricular tachycardia. *Circulation* **136**, 2491–2507 (2017).

10. Andreu, D. *et al.* A QRS axis–based algorithm to identify the origin of scar-related ventricular tachycardia in the 17-segment American Heart Association model. *Heart Rhythm* **15**, 1491–1497 (2018).

11. Graham, A. J. *et al.* Evaluation of ECG imaging to map hemodynamically stable and unstable ventricular arrhythmias. *Circulation: Arrhythmia and Electrophysiology* **13**, e007377 (2020).
